# Belzutifan-Associated Hypoxia: A Review of the Novel Therapeutic, Proposed Mechanisms of Hypoxia, and Management Recommendations

**DOI:** 10.3390/ijms26157094

**Published:** 2025-07-23

**Authors:** John Kucharczyk, Anshini Bhatt, Laura Bauer, Minas Economides

**Affiliations:** 1 Department of Oncology and Hematology, Perlmutter Cancer Center at NYU Langone Hospital—Long Island, Mineola, NY 11501, USA; 2 Department of Genitourinary Medical Oncology, Perlmutter Cancer Center, NYU Langone Health, New York, NY 10016, USA; anshini.bhatt@nyulangone.org (A.B.); laura.bauer@nyulangone.org (L.B.); minas.economides@nyulangone.org (M.E.)

**Keywords:** renal cell carcinoma, belzutifan, hypoxia-inducible factor-2α (HIF-2α), hypoxia, adverse events

## Abstract

Belzutifan is a hypoxia-inducible factor-2α (HIF-2α) inhibitor that received FDA approval in 2021 for treating cancers resulting from von Hippel-Lindau (VHL) disease, including clear cell renal cell carcinoma (ccRCC), followed by approval in 2023 for sporadic ccRCC that has progressed through multiple lines of therapy. HIF-2α is a promising drug target, as VHL is commonly inactivated in ccRCC, which results in HIF-2α-mediated signaling that is considered central to tumorigenesis. Belzutifan has demonstrated efficacy in clinical trials in the first-line and subsequent line settings, and in combination with tyrosine kinase inhibitors. Despite being overall well tolerated, belzutifan has a distinct safety profile because of its unique mechanism of action. Anemia was the most common adverse event observed in clinical trials and is considered an on-target effect. Hypoxia is also frequently observed and commonly results in dose reductions, treatment discontinuation, and supplemental oxygen use. This review summarizes the rates of hypoxia seen in clinical trials of belzutifan in ccRCC. As the cause of hypoxia is not well understood, this review also discusses possible mechanisms of hypoxia based on preclinical studies of the HIF pathway and HIF-2α inhibitors. Finally, this review proposes monitoring and management recommendations for clinicians prescribing belzutifan to ccRCC patients.

## 1. Introduction

The von Hippel-Lindau (VHL) gene plays a central role in the pathogenesis of clear cell renal cell carcinoma (ccRCC) and is inactivated in approximately 90% of ccRCC tumors [1]. The VHL protein (pVHL) is a key regulator of the hypoxia-inducible factor (HIF) family of transcription factors, primarily HIF-1α and HIF-2α, which have both overlapping and distinct biological functions in ccRCC progression. Three different HIF isoforms have been identified, with HIF-1α and HIF-2α being the most well-studied. Each has some common and subtype-specific targets, and they have distinct roles in the pathogenesis of ccRCC [2,3]. While HIF-1α is ubiquitously expressed, HIF-2α is mostly expressed in endothelial, lung, renal, and hepatic cells [4]. Loss of VHL results in a “pseudo-hypoxic” state increasing the expression of HIF-1α and HIF-2α, which results in pathway activations, eventually promoting ccRCC growth and progression [5]. This understanding has been instrumental in the development of HIF-2α inhibitors as a targeted therapeutic approach in ccRCC. Belzutifan is the first and only HIF-2α inhibitor approved by the US Food and Drug Administration (FDA) for ccRCC treatment [6]. It was first approved in VHL disease, an inherited condition caused by mutations in VHL that result in ccRCC and other malignancies, and later for all ccRCC patients that have progressed after treatment with both an immune checkpoint and a vascular endothelial growth factor (VEGF) inhibitor [6,7]. In clinical trials, hypoxia was the second most common adverse event associated with belzutifan, leading to treatment discontinuation, supplemental oxygen use, or hospitalization in a significant subset of patients [8,9]. Hypoxia is a unique toxicity of belzutifan that has not been reported at clinically significant rates in the registration trials of VEGF TKIs for RCC [10,11]. The exact mechanism underlying belzutifan-induced hypoxia is not entirely understood but is felt to be related to HIF-2α’s role in normal physiology within the kidney and lungs. This review explores the mechanism of action of belzutifan, its clinical development and evidence for use in metastatic ccRCC, potential mechanisms contributing to hypoxia, and recommendations for monitoring patients undergoing treatment.

## 2. Mechanism of Action

In normoxia, oxygen-dependent prolyl hydroxylases (PHDs) hydroxylate proline residues within the oxygen-dependent domain of hypoxia-inducible factor-2α (HIF-2α) to create a recognition site for the VHL protein (pVHL) [12]. The pVHL E3 ubiquitin ligase complex recognizes prolyl-hydroxylated HIF-2α and targets it for ubiquitylation and rapid proteasomal degradation [13]. In hypoxic conditions, there is insufficient oxygen to activate PHDs; HIF-2α remains unmodified, and thus, pVHL cannot facilitate its degradation [14]. Excess HIF-2α is known to promote angiogenesis and tumor invasion, for example, through upregulation of hypoxia-induced genes such as VEGF and erythropoietin by heterodimerizing with aryl hydrocarbon receptor nuclear translocator (ARNT) to form the transcription factor hypoxia-inducible factor 1 (HIF-1) [15,16].

For a time, HIF-2α was considered undruggable [17]. However, an internal cavity within the PAS-B domain was found to be amenable to ligand binding [18]. This discovery enabled researchers from Peleton Therapeutics to develop the small molecule inhibitor PT2385 that allosterically blocks the dimerization of HIF-1α/2α to ARNT. PT2385 inhibited the expression of HIF-2α-dependent genes associated with tumor growth, including VEGF-A, PAI-1, and Cyclin D1 in ccRCC cell lines, and induced tumor regression in xenograft models [19].

## 3. Clinical Development

### 3.1. Phase I Studies

A phase I dose-escalation trial of PT2385 was conducted in pretreated ccRCC patients and showed a favorable safety profile and disease activity [20]. However, because of suboptimal pharmacokinetics, a second-generation HIF-2α inhibitor, PT2977 (later named MK-6482 and then belzutifan), was developed [14]. Belzutifan was about tenfold more potent than PT2385 in xenograft models [14]. In the first-in-human phase I study, LITESPARK-001, 120 mg daily was determined to be the recommended phase II dose [21]. The ccRCC cohort included 55 patients who were heavily pretreated; the objective response rate (ORR) with belzutifan was 25% (all partial responses), and the median progression-free survival was 14.5 months (Table 1) [22]. In the ccRCC cohort, the most common grade 3 or higher adverse events were anemia (27%) and hypoxia (16%) [22]. Anemia is considered an on-target effect, as erythropoietin is one of the proteins upregulated by excess HIF-2α. Of the 17 patients who experienced hypoxia, 11 received supplemental oxygen, six required a dose reduction, two required an interruption, and two required treatment discontinuation.

### 3.2. Phase II Studies

Von Hippel-Lindau disease is an autosomal dominant inherited disorder caused by germline mutations of VHL and is associated with an approximately 70% lifetime risk of developing ccRCC [23]. VHL disease patients are at increased risk of malignancies in addition to RCC, most commonly central nervous system (CNS) hemangioblastoma (60–80% lifetime risk) and pancreatic neuroendocrine tumors (PNETs, 9–17% lifetime risk) [24]. It was posited that belzutifan would be particularly effective in the VHL disease population, as dysfunctional VHL results in constitutive activation of HIF-dependent transcription factors. The LITESPARK-004 phase II trial evaluated belzutifan use in 61 VHL disease patients with RCC, the majority of whom had simultaneous central nervous system (CNS) hemangiomas or pancreatic neuroendocrine tumors [8]. In RCC tumors, an ORR of 49% with a median time to response of 8.2 months was observed. Only one of the 61 patients in the study experienced hypoxia, compared to 31% observed in LITESPARK-001. The patient experienced a transient grade 3 hypoxia; it was resolved with a 1-week dose interruption and then a dose reduction to 80 mg daily [8]. This dose modification is listed on the FDA prescribing information and advised for the management of adverse events, although data on its efficacy does not exist [25]. However, such a dose modification represents the practice used in all trials studying belzutifan. Regarding CNS hemangioblastomas and PNETs, confirmed responses were seen in 15 of 50 (30%) and 20 of 22 (91%) of patients, respectively [22]. These findings led to the first approval for belzutifan use by the US FDA in August 2021, which was for VHL disease patients with ccRCC, CNS hemangioblastoma, and PNETs [7].

The phase II LITESPARK-013 randomized 154 pretreated ccRCC patients to receive either 120 mg or 200 mg of belzutifan [26]. There was no difference in response rates between the 120 mg and 200 mg cohorts; ORR was 23.7% vs. 23.1%, respectively. Among the 120 mg cohort, hypoxia was reported in 18 (23.7%) patients, with 16 (21.1) reporting grade 3 or higher.

Cabozantinib is a multikinase TKI that targets several receptors in the angiogenesis pathway, including VEGF, and is approved for mRCC treatment both in combination with an immune checkpoint inhibitor and as a single agent. A phase II study combining belzutifan and cabozantinib was inspired by the hypothesis that combining a HIF-2α inhibitor and VEGF TKI would have a synergistic antitumor effect [27]. The combination was given to 52 ccRCC patients who had previously received an immune checkpoint inhibitor and up to two prior systemic treatments; 16 (30.8%) had an objective response, including one (2%) who had a complete response and 15 (29%) who had partial responses. The most common grade 3 or higher adverse event was hypertension (27%); two (4%) patients experienced hypoxia, both of which were grade 3.

**Table 1:** Response rates and adverse events of belzutifan trials (ORR, objective response rate).

Study ID (NCT Trial Number)	Treatment Arm	Number of Patients	Patient Population	ORR (%)	Most Common Grade ≥ 3 (%)	Any Grade Hypoxia (%)	Grade ≥ 3 Hypoxia (%)
LITESPARK001 (NCT02974738) [22,28]	Belzutifan for ccRCC	55	ccRCC previously treated with ≥1 therapy	25	Anemia (27)	17 (31)	9 (16)
LITESPARK-004 (NCT03401788) [8,29]	Belzutifan	61	ccRCC with VHL disease, not all pretreated	49	Anemia and hypertension (8)	1 (2)	1 (2)
LITESPARK-013 (NCT04489771) [26]	Belzutifan 120 mg 200 mg	76 78	ccRCC previously treated with one to three therapies	23.7 23.1	Hypoxia (21.1) Anemia (26.9)	18 (23.7) 21 (26.9)	16 (21.1) 17 (21.8)
LITESPARK-005 (NCT04195750) [9,30]	Belzutifan	374	ccRCC previously treated with immune and antiangiogenic therapies	21.9	Anemia (32.5)	54 (14.5)	39 (10.5)
LITESPARK-003 (NCT03634540) [27,31]	Belzutifan + Cabozantinib	52	ccRCC previously treated with immune therapy	30.8	Hypertension (27)	2 (4)	2 (4)

### 3.3. Phase III Studies

Subsequently, the LITESPARK-005 phase III trial was conducted comparing belzutifan to everolimus in 746 sporadic unresectable or metastatic ccRCC patients who had disease progression after treatment with an immune checkpoint inhibitor and a VEGF inhibitor (received in sequence or combination) [9]. A confirmed objective response was observed in 21.9% of patients in the belzutifan group versus 3.5% in the everolimus group. The belzutifan group experienced a significantly prolonged progression-free survival (PFS) and a trend to prolonged overall survival, although data were immature at publication. The percentage of patients experiencing adverse events from any cause was very similar between the belzutifan and everolimus groups [9]. Again, anemia was the most common adverse event in the belzutifan group (82.8% any grade, 32.5% grade 3). Hypoxia was seen in 54 patients (14.5%), with 39 (10.5%) experiencing grade 3 or higher; 38 (70%) patients in the belzutifan group that were hypoxic required supplemental oxygen. The median time to the onset of hypoxia was 30.5 days (range 1 day to 21.1 months). In December 2023, belzutifan with a recommended dose of 120 mg was approved by the US FDA in mRCC regardless of VHL disease status in patients that have received an immune checkpoint inhibitor and a VEGF inhibitor [6].

A pooled analysis of the 576 patients in the LITESPARK-001, LITESPARK-004, LITESPARK-0013, and LITESPARK-005 found that 217 (37.7%) patients experienced a grade 3 or higher adverse event [32]. Hypoxia was reported in 94 patients (16.3%); among them, 66 (70.2%) were treated with supplemental oxygen. Grade 3 or higher hypoxia occurred in 57 patients (9.9%). The median time to resolution of hypoxia was 11 days, speaking to its transient nature. Other adverse events in the pooled analysis were fatigue (42.7% any grade), nausea (21.4% any grade), and dizziness (17.9% any grade). However, these uncommonly led to dose interruptions or discontinuation, 2.6% and 0.2% from fatigue, 2.4% and 0.2% from nausea, and 1.6% and 0.2% from dizziness, respectively. A higher rate of hypoxia was seen in a recent retrospective study of “real-world” belzutifan use. In twenty-two patients, eight (36%) experienced hypoxia, six required supplemental oxygen, and two were hospitalized because of hypoxia [33].

### 3.4. Ongoing Clinical Trials

There are active phase III clinical trials investigating belzutifan in ccRCC in the adjuvant, first-line, and subsequent line settings (Table 2). A study of adjuvant treatment, NCT05239728, compares the combination of pembrolizumab and belzutifan versus pembrolizumab and placebo after nephrectomy [34]. One year of adjuvant pembrolizumab is approved for high-risk ccRCC patients based on the results of the phase III KEYNOTE-564 trial, which demonstrated an improvement in disease-free survival versus placebo, and later an overall survival advantage [35,36].

A three-armed phase III study in the first-line setting compares adding belzutifan to the doublet of pembrolizumab and lenvatinib versus pembrolizumab and the CTLA-4 inhibitor, quavonlimab, versus pembrolizumab and lenvatinib [37]. Pembrolizumab plus lenvatinib was approved by the FDA in the first-line setting in 2021 based on the phase III CLEAR study, where it demonstrated superior ORR, PFS, and OS compared to sunitinib [38]. The efficacy and safety of the triplet of belzutifan, pembrolizumab, and lenvatinib will be compared to the standard of care doublet.

An additional phase III trial is comparing the combination of lenvatinib and belzutifan versus single-agent cabozantinib in mRCC patients that have progressed on an immune checkpoint inhibitor [39]. The combination of belzutifan and cabozantinib has shown activity in a previously mentioned phase II study of ccRCC patients that had progressed after PD-1 therapy [27]. It is hoped that belzutifan and lenvatinib will have a synergistic antitumor effect by simultaneously suppressing HIF-2α-induced oncogene upregulation while also disrupting downstream signaling of growth factors, including VEGF. Lenvatinib was previously approved in the second-line setting in combination with everolimus in 2016, when it demonstrated superior PFS compared to everolimus monotherapy [40].

**Table 2:** Active or recently completed clinical trials studying belzutifan in RCC.

Trial Number NCT Identifier	Phase	Intervention	Setting	Line of Therapy	Recruitment Status
**Phase I Studies**					
NCT06234605 [41]	I	HC-7366 Monotherapy versus HC-7366 + Belzutifan	Locally advanced (unresectable) or metastatic clear cell renal cell carcinoma (ccRCC)	1st line	Recruiting
NCT05030506 [42]	I	Belzutifan + Lenvatinib Experimental Arm: Belzutifan + Lenvatinib + Pembrolizumab	Locally advanced (unresectable) or metastatic clear cell renal cell carcinoma (ccRCC)	2nd line Experimental Arm: 1st line	Active, Not Recruiting
NCT04626479 [43]	I	Pembrolizumab/Quavonlimab + Lenvatinib versus Favezelimab/Pembrolizumab + Lenvatinib versus Pembrolizumab + Belzutifan + Lenvatinib versus Pembrolizumab + Lenvatinib versus Vibostolimab/Pembrolizumab + Belzutifan	Metastatic ccRCC	1st line	Active, Not Recruiting
NCT02974738 [22,28]	I	Belzutifan Monotherapy with ccRCC and advanced solid tumors	Locally advanced ccRCC or metastatic solid tumors	1 line+	Active, Not Recruiting
NCT02293980 [44]	I	MK-3795, formerly called PT2385 versus MK-3795 + Nivolumab + Belzutifan versus MK-3795 + Cabozantinib	Advanced or metastatic clear-cell renal cell carcinoma (ccRCC)	1 line+	Active, Not Recruiting
NCT04846920 [45]	I	Belzutifan 160 mg BID Belzutifan 160 mg TID Belzutifan 200 mg TID Belzutifan 120 mg QD	Advanced clear-cell renal cell carcinoma (ccRCC)	2 lines+	Active, Not Recruiting
NCT04626518 [46]	I	Coformulation Pembrolizumab/Quavonlimab versus Coformulation Favezelimab/Pembrolizumab versus Pembrolizumab + MK-4830 versus Pembrolizumab + Belzutifan versus Belzutifan + Lenvatinib versus Pembrolizumab + Lenvatinib	Advanced clear-cell renal cell carcinoma (ccRCC)	2 lines+	Active, Not Recruiting
NCT05468697 [47]	I/II	Belzutifan Monotherapy versus Belzutifan + Palbociclib	Advanced clear-cell renal cell carcinoma (ccRCC)	2 lines+	Recruiting
**Phase II Studies**					
NCT03634540 (MK-6482-003) [27,31]	II	Belzutifan + Cabozantinib (Treatment Naïve) versus Belzutifan + Cabozantinib (Prior Immunotherapy)	Advanced or metastatic clear-cell renal cell carcinoma (ccRCC)	1st/2nd line	Active, Not Recruiting
NCT03108066 [48]	II	MK-3795 (PT2385)	Von Hippel-Lindau (VHL) disease-associated clear cell renal cell carcinoma (ccRCC)	1st line	Completed
NCT03401788 (MK-6482-004) [8,29]	II	Belzutifan Monotherapy	VHL disease + RCC tumor	1st line	Active, Not Recruiting
NCT04489771 [26,49]	II	Belzutifan 200 mg versus Belzutifan 120 mg	Advanced or metastatic clear-cell renal cell carcinoma (ccRCC)	2nd line	Active, Not Recruiting
**Phase III Studies**					
NCT05239728 [34]	III	Belzutifan + Pembrolizumab versus Placebo + Pembrolizumab	Adjuvant treatment of clear cell renal cell carcinoma (ccRCC) post-nephrectomy	1st line	Active, Not Recruiting
NCT05899049 [50]	III	Pembrolizumab + Belzutifan + Lenvatinib versus Pembrolizumab/Quavonlimab + Lenvatinib	Advanced clear cell renal cell carcinoma (ccRCC)	1st line	Active, Not Recruiting
NCT04736706 [37]	III	Pembrolizumab + Belzutifan + Lenvatinib or Pembrolizumab/Quavonlimab + Lenvatinib versus Pembrolizumab plus Lenvatinib	Advanced clear cell renal cell carcinoma (ccRCC)	1st line	Active, Not Recruiting
NCT04586231 [39]	III	Belzutifan + Lenvatinib versus Cabozantinib	Locally advanced (inoperable) or metastatic clear cell renal cell carcinoma (RCC)	1st/2nd line	Active, Not Recruiting
NCT04195750 [9,30]	III	Belzutifan versus Everolimus	Advanced or metastatic clear-cell renal cell carcinoma (ccRCC)	2 lines+	Active, Not Recruiting

## 4. Mechanism of Belzutifan-Induced Hypoxia

**Figure 1:**
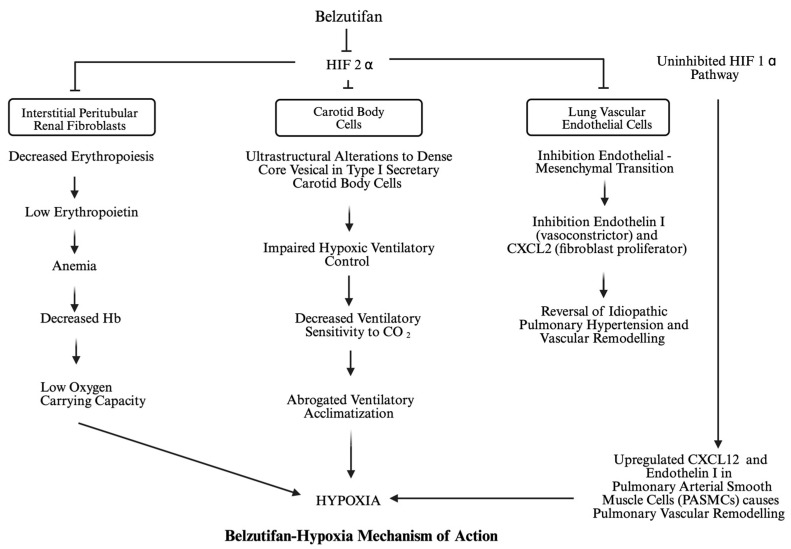
Proposed mechanisms of hypoxia from HIF-2α inhibition. Created in BioRender. Bhatt, A. (2025) https://BioRender.com/mlzyv8u. Belzutifan’s HIF-2α inhibition decreases oxygen carrying capacity by inducing anemia. It additionally interferes with the carotid body’s ability to adjust ventilatory control in response to both hypoxia and elevated CO_2_. HIF-2α inhibition in lung vascular endothelial cells inhibits or reverses vascular remodeling, so this is not likely a cause of hypoxia. However, uninhibited HIF-1α activation can lead to hypoxia through vascular remodeling.

The mechanism of hypoxia from belzutifan administration is likely multifactorial and not completely understood (Figure 1). The HIF pathway is involved in many aspects of normal physiology, and so it is likely that a HIF-2α inhibitor is affecting the function of non-cancerous tissues. Erythropoietin is secreted by renal interstitial fibroblasts and regulated by HIF-2α. One potential cause of hypoxia is anemia resulting from HIF-2α inhibition and thus reduced oxygen-carrying capacity [51].

Hypoxia, however, can occur independent of the occurrence of anemia. HIF-2α is known to mediate the hypoxic ventilatory response, primarily in the carotid body [52]. Cheng et al. administered PT2385 to wild-type and Epas1 (the gene encoding HIF-2α)-mutated mice [53]. At PT2385 doses that have efficacy against ccRCC, wild-type mice had an abrogated or absent response to sustained hypoxia, which included gene expression changes, ultrastructural alterations to dense core vesicles in type I secretory cells, and multilineage cell proliferation. This mechanism was preserved in Epas1-mutated mice, indicating that PT2385’s interference with the hypoxia response is an on-target effect. The reduced sensitivity of PT2385-treated mice to carbon dioxide in this setting is also consistent with an action of PT2385 on ventilatory acclimatization to hypoxia. In summary, these findings reveal that treatment with PT2385 exerts striking effects on ventilatory control, essentially ablating the enhanced ventilatory sensitivity, or ventilatory acclimatization that occurs during sustained exposure to hypoxia [52].

Dysregulation of the HIF pathway is known to result in pulmonary hypertension. Chuvash polycythemia, endemic to the Chuvash region of Russia, is an inherited syndrome caused by a homozygous germline loss-of-function mutation in VHL, specifically VHLR200W, that results in polycythemia and pulmonary hypertension [54]. Similarly, an inherited activating mutation of HIF-2α has also been associated with polycythemia and pulmonary hypertension [55]. HIF-2α is known to be expressed in lung vascular endothelial cells (LVECs) [4]. Excess HIF-2α has been shown to induce the endothelial-to-mesenchymal transition of LVECs that ultimately causes hypoxia-induced pulmonary hypertension through vascular remodeling [56]. This implication of HIF-2α in the pathogenesis of pulmonary hypertension led to Ghosh et al. testing belzutifan in mouse models of pulmonary hypertension [57]. Belzutifan effectively reversed polycythemia and pulmonary hypertension in multiple mouse models that disrupt the HIF pathway, including mice homozygous for a VHLR200W mutation. Similarly, Dai et al. treated endothelial cells from idiopathic pulmonary arterial hypertension (PAH) patients and rodent models of PAH with a selective HIF-2α inhibitor [58]. HIF-2α inhibition resulted in reversal of pulmonary vascular remodeling and right ventricular hypertrophy and promoted survival in multiple rodent models of PAH.

These results are seemingly paradoxical to the association of belzutifan and hypoxia in patients receiving it for the treatment of metastatic RCC. In the mouse models of HIF pathway alteration treated with belzutifan by Ghosh et al., pulmonary hypertension seems to have been caused by increased endothelin-1, a HIF target and vasoconstrictor, and Cxcl-12, a fibroblast proliferation and idiopathic pulmonary fibrosis promoter. The authors showed that belzutifan reduced the expression of both endothelin-1 and Cxcl-12, and proposed this as the mechanism of pulmonary hypertension reversal. Perhaps in metastatic RCC patients who presumably do not have genetic alterations to the HIF pathway in their LVECs, belzutifan alters the balance of vasoconstriction and fibrosis signals, resulting in transient hypoxia.

Another mechanism of pulmonary hypertension is the uninhibited and upregulated HIF-1α pathway during hypoxia in patients receiving treatment with belzutifan. Notably, in both cancer cells and ECs (endothelial cells), HIF-1α accumulates earlier during hypoxia, and its levels decrease more rapidly than HIF-2α during prolonged hypoxia [59]. In addition, several studies have demonstrated that both HIF isoforms can even display opposing roles in vivo, for example, in renal cell carcinoma growth and metastasis formation [60]. HIF-1α-driven CxCl12 expression in PAECs (pulmonary artery endothelial cells) regulates bone marrow-derived progenitor cell increases in PAH patients. Endothelin 1 stabilizes HIF-1α, promoting glycolytic switch and pulmonary vascular remodeling [61]. HIF-1α has been suggested to represent the response to acute hypoxia, whereas HIF-2α is the predominant subunit to chronic exposure to low oxygen that occurs at high altitudes [62,63]. In the trial of VHL disease patients, LITESPARK-004, only one (1.6%) of 61 patients experienced hypoxia, compared to 14.5% of patients in the LITESPARK-005 [8]. This difference in hypoxia incidence is perhaps because the VHL disease population was younger and had better performance statuses [8]. The reduced incidence of hypoxia may also be due to differences in HIF signaling in pulmonary tissue after belzutifan treatment between VHL-mutated and wild-type VHL patients. Based on current clinical trial data, belzutifan does not seem to cause the permanent hypoxia that would be expected in pulmonary vascular remodeling or fibrosis.

The variability in tissue hypoxia observed during HIF-2α inhibition may, in part, be explained by the compensatory activity of HIF-1α. Although HIF-1α and HIF-2α are structurally homologous, they exhibit distinct and sometimes opposing regulatory functions—a phenomenon termed the “HIF switch” [64]. Preclinical studies using the selective HIF-2α inhibitor PT2385 demonstrated the suppression of HIF-2α–dependent genes such as VEGF-A, PAI-1, cyclin D1, CXCR4, and GLUT1, with minimal effects on HIF-1α–regulated transcripts, suggesting an intact and potentially compensatory HIF-1α axis [19]. Furthermore, HIF-2α drives a positive feedback loop via transcriptional upregulation of PHD3 (EGLN3), which stabilizes HIF2A mRNA and supports sustained expression. Belzutifan, by inhibiting HIF-2α, disrupts this autoregulatory loop, leading to decreased PHD3 expression and attenuation of HIF-2α transcriptional activity [65].

Beyond direct effects on oxygen metabolism, HIF inhibition also intersects with immune regulation and microbial homeostasis. Hypoxia is a known modulator of immune responses, with HIF-1α promoting pro-inflammatory functions through enhanced glycolytic activity in macrophages and T cells, while HIF-2α supports anti-inflammatory phenotypes, including IL-10 expression and M2 macrophage polarization [66]. In mouse models, HIF-2α inhibition altered tumor-associated macrophage (TAM) profiles and reduced myeloid infiltration [66,67]. These immune changes may, in turn, impact local oxygen tension and feed back onto microbial ecosystems, especially in gut and pulmonary tissues, where oxygen availability is tightly coupled to microbiome composition. While direct clinical data on microbiome or immune shifts during belzutifan treatment are lacking, emerging preclinical evidence supports a microbiome–immune–hypoxia axis, which may influence both treatment response and toxicity.

## 5. Monitoring of Patients with Belzutifan

Belzutifan can cause severe hypoxia that may result in supplemental oxygen, hospitalization, or treatment discontinuation. Thus, patients should be advised to contact their physician immediately if they experience signs or symptoms of hypoxia, including dizziness, shortness of breath, or chest pain. Given that one potential mechanism of belzutifan-induced hypoxia is a reduced hypoxic ventilatory response, providers should have a low threshold to prescribe supplemental oxygen and a pulse oximeter, as their ability to compensate for hypoxia with increased respiratory rate may be diminished. Particular attention should be paid to reported dyspnea and oxygen saturation at each clinic visit. In the case of exercise-induced hypoxia, it is recommended to withhold belzutifan until it has resolved and then resume at either the same or a reduced dose [25]. For hypoxia at rest, treatment interruption should be considered, and resuming at a reduced dose when the hypoxia has resolved or permanent discontinuation are reasonable.

Presently, there is no standard of practice that exists for monitoring patients on belzutifan. The FDA recommends general monitoring with periodic oxygen saturation checks and lab work to assess for anemia at all follow-up visits [25]. Given the expanded use of this medication, providers should attempt to standardize the monitoring of these patients, starting with establishing baseline oxygen saturation levels prior to initiation. It is advisable to follow patients especially closely during the first month of treatment, as that is when hypoxia most commonly occurs, with or without symptoms. At minimum, patients on the drug should be seen monthly or more frequently, based on provider discretion.

Many hospital systems are developing technology that allows patients to utilize home pulse oximetry monitors that communicate directly to their electronic medical record. These applications can push notifications to members of the oncology team directly to alert them of changes in oxygen levels. Borderline oxygen levels, 90–94%, should trigger an in-person assessment with labs to check for anemia. It is advisable for patients to log home monitoring values at least once a day. With improved outpatient monitoring, many hospital admissions and treatment interruptions may be avoided. Additionally, patients who require supplemental oxygen can be identified and intervened on earlier.

## 6. Conclusions

Belzutifan is a promising new addition to the armamentarium of systemic treatment options for metastatic ccRCC. It is approved for use in ccRCC patients after progression on an immune checkpoint inhibitor and VEGF inhibitor, and in VHL disease patients with ccRCC, CNS hemangioblastomas, or PNETs not requiring immediate surgery; it is currently being investigated in the front-line setting.

As the use of this medication grows in the community, it is essential for providers to understand its unique side effects and optimal management strategies. Belzutifan is effective in RCC by inhibiting HIF-2α and thus interfering with “pseudo-hypoxic” signaling pathways known to be central to RCC formation and progression. Early data on belzutifan use indicate that it causes severe hypoxia in a subset of patients that can result in treatment discontinuation, a requirement for supplemental oxygen, or hospitalization. Although the mechanism for this adverse effect is not entirely understood, it is perhaps related to HIF-2α’s role in normal physiology, including erythropoiesis and the hypoxic ventilatory response. Educating patients about the risk of hypoxia before starting treatment is essential. We recommend frequent monitoring, particularly during the first two months, and advocate for the use of home oxygen saturation devices.

## Abbreviations

The following abbreviations are used in this manuscript:

VHL	von Hippel-Lindau
ccRCC	Clear cell renal cell carcinoma
HIF-1α	Hypoxia-inducible factor-1α
HIF-2α	Hypoxia-inducible factor-2α
FDA	Food and Drug Administration
VEGF	Vascular endothelial growth factor
CNS	Central nervous system
PNET	Pancreatic neuroendocrine tumors
TKI	Tyrosine kinase inhibitor
ORR	Overall response rate
PFS	Progression-free survival
OS	Overall survival
